# The value of the qualitative method for adaptation of a disease-specific quality of life assessment instrument: the case of the Rheumatoid Arthritis Quality of Life Scale (RAQoL) in Estonia

**DOI:** 10.1186/1477-7525-2-69

**Published:** 2004-12-04

**Authors:** Marika Tammaru, Judit Strömpl, Kadri Maimets, Ele Hanson

**Affiliations:** 1 Department of Internal Medicine, Faculty of Medicine, University of Tartu, Puusepa 6, Tartu, 51014, Estonia; 2 Department of Social Policy and Social Work, Faculty of Social Sciences, University of Tartu, Estonia

## Abstract

**Background:**

Due to differences in current socio-economical situation and historically shaped values, different societies have their own concepts of high-quality life. This diversity of concepts interferes with quality of life (Qol) research in health sciences. Before deciding to apply a Qol assessment tool designed in and for another society, a researcher should answer the question: how will this instrument work under the specific circumstances of my research. Our study represents an example of the utilization of qualitative research methods to investigate the appropriateness of the Rheumatoid Arthritis Quality of Life Scale (RAQol) for the assessment of Qol in Estonian patients.

**Methods:**

Semi-structured interviews were conducted with the rheumatoid arthritis (RA) patients of Tartu University Hospital and these were analyzed using the principles of the grounded theory.

**Results:**

We described the significance of the questionnaire's items for our patients and also identified topics that were important for the Qol of Estonian RA patients, but that were not assessed by the RAQol. We concluded that the RAQol can be successfully adapted for Estonia; the aspects of Qol not captured by the questionnaire but revealed during our study should be taken into account in future research.

**Conclusions:**

Our results show that qualitative research can successfully be used for pre-adaptation assessment of a Qol instrument's appropriateness.

## Background

With this article we are introducing our experience of how qualitative research can be utilized in the process of adapting of a quality of life (Qol) assessment instrument. We will argue the unique benefits of the qualitative method for assuring the validity of an adapted measure.

Qol cannot be treated as something uniform and stable. One reason for its variability in time and space is the social nature of the "quality". Different societies with their current socio-economic situation, values and traditions, carry their own, to certain degree dissimilar understandings of high-quality life. Therefore Qol can be seen as a social construct. For further discussion on Qol domains see Schalock, 2004 [1].

Inclusion of Qol assessment into a set of health outcome measures is, regarding the achievements in improving the survival of chronically ill patients, well justified. But the social complexity of the construct means that its evaluation is also complex compared to traditional outcome measures e.g. symptoms or results of lab tests. Restricting Qol assessment to condition-bound groups (disease-specific Qol) permits potential influences on everyday life to be homogenized; problems with the various individual significances of these condition-characteristic impacts remain. Different Qol concepts in health sciences try to improve assessment's generalizability. Functionalistically oriented health-related Qol concentrates on restrictions in performance of everyday activities [2]. Although this approach allows presumably quite characteristic impacts of health condition or disease to be described and compared, it does omit several, mostly social and cultural, factors. Without considering these influences the detailed specification of Qol remains incomplete. The needs-based Qol model proceeds from the motivationalists' idea about universal human needs and defines Qol as a level of satisfaction of these needs [3]. Although universal in theory, this approach still has its imperfections regarding practical assessment: ways of fulfillment of even universal needs depend on the possibilities offered by society; measurement of these needs' satisfaction is accessible through rating their fulfillment acts; thereby, assessment of Qol cannot be liberated from time and space dimensions.

Before deciding to use an existing instrument for Qol assessment, a researcher should answer the question: how will this questionnaire work in the particular circumstances of the research? Even an instrument that has performed excellently in the country of origin can loose some validity when applied in a different social context. There are three categories of topics that should be recognized for every Qol instrument: first, those important for the patients being investigated, and already assessed by the instrument ; second, those unimportant (or not so important) for the patients being investigated, but still assessed by the instrument; and third, those important the for the patients being investigated, but not assessed by the instrument. It is evident that an appropriate questionnaire consists mostly of the first category items, the content of the second category items is minimized and all topics important for patients are included.

The items belonging to the two first categories can be determined during a standard adaptation process. The drawback of this approach is the amount of adaptation work that usually has to be done before receiving evidence about the merits of the questionnaire. Delimiting of the third category requires a deeper insight into the society-specific determinants of Qol.

Because of the smallness of Estonia (population about 1.4 million) studies involving only our patients may demonstrate a lack of statistical power; also some restrictions concerning scientific potential and funding should be admitted. Therefore a promising choice for Estonian clinical and health sciences can be seen in cooperative research projects. Reasoned selection, judicious adaptation and application of internationally approved assessment instruments could be crucial for success. As a part of the Soviet Union, Estonia stayed isolated from Europe for years and quite strong ideological pressure was brought to bear on the citizens. After establishing independence in the 1990s, Estonian society has undergone abrupt ideological and economic changes. A researcher working in the Qol assessment field should take into account the suspected impact of these factors on understanding life quality among the local population.

Our work offers an example of the application of qualitative interviews for exploring the essence of quality of everyday life for Estonian rheumatoid arthritis (RA) patients. We will illustrate the evaluation of the three topic categories using an example of RA specific Qol assessment scale.

### The questionnaire of interest

Rheumatoid Arthritis Quality of Life Scale (RAQol) was developed in the 1990s as an outcome measure to assess the impact of RA and its treatment on QoL. The content was derived from interviews with 50 RA patients conducted simultaneously in Great Britain and the Netherlands [4,5]. The theoretical basis for the RAQoL is the needs-based model of QoL. The basic list of needs the model considers to be crucial to Qol had been published earlier [3].

#### List of needs

• food, drink, sleep, activity, sex, pain avoidance

• warmth, shelter, security, safety, freedom from fear, stability

• affection, love, physical contact, intimacy, attachment, communication, sharing experiences, sharing goals, affiliation

• curiosity, exploration, play, stimulation, enjoyment, creativity, meaningfulness

• identity, status, recognition, approval, appreciation, usefulness to others, respect, competence, self esteem, mastery, achievement, power, independence, freedom

• time structure

• self actualization

As a RA specific instrument, the RAQol was designed to assess the fulfillment of only those needs whose importance for RA patients emerged during the interviews. The original wording from the interviews was retained as much as possible. The final RAQoL is a 30-item measure where each item is in the form of a simple statement to which patients indicate whether or not it is true for them at that moment. The following example of the RAQol items demonstrates the wide range of everyday areas covered by the instrument: self-care, different indoor and outdoor activities, emotions and conditions, and interpersonal relations.

#### An example of the RAQol items

• Item 18. I have problems taking a bath/shower

• Item 7. Jobs about the house take me a long time

• Item 6. I find it difficult to walk to the shops

• Item 16. I often get depressed

• Item 12. I find it hard to concentrate

• Item 13. Sometimes I just want to be left alone

• Item 29. I avoid physical contact

An affirmed item indicates an adverse quality of life. Each item on the RAQoL is scored '1' for the affirmed statement or '0' for the disaffirm statement. All item scores were summed to form a total score ranging from 0 (good QoL) to 30 (poor QoL). The original RAQol exists in two versions – UK English and Dutch; currently seven language adaptations of the RAQoL are available for use. Excellent test-retest reliability, internal consistency and construct validity of the original instrument and its versions has been demonstrated [6-10]. The RAQoL provides a valuable tool for assessing the impact of RA on QoL in international clinical trials and other research studies. For a full list of the RAQol items see de Jong *et al. *1997 [5].

### Goal and research questions

The lack of an instrument for systematic assessment of the Qol of RA patients in Estonia raised the question of the feasibility of adapting the RAQol. Our task was to assess the value of application of the qualitative method for describing the appropriateness of the questionnaire before the adaptation. For studying the three above-described topic categories in connection with the RAQol, we formulated the following two research questions:

• Are the RAQol's topics important for our patients?

• What else do our patients find to be significant in connection with their everyday life quality?

## Method

### The choice of method and structure of interview

We decided to apply thematic analysis following the principles of the grounded theory. Our choice was determined by the second research question. To answer it, the analysis had to be guided by the data itself, to enable new motifs and theories to spring up. The grounded theory, evolved by Barney G Glaser and Anselm L Strauss in the 1960s [11] and developed later by them and other scholars [12,13], offers a systematic approach for analyzing qualitative data using both inductive (open and axial coding, generation of core categories) and deductive (selective coding and theoretical/selective sampling) approaches in data processing [12]. The prerequisite for this inductive-deductive data handling is the simultaneous running of the processes of data collecting, coding and analysis [13].

For data collection we decided to apply individual interviews, which we considered to be the method of choice for investigating RA patients' perceptions of quality of their everyday lives. An alternative focus-group method was rejected because of the possible intimacy of some topics, which might have been difficult to discuss openly in a group setting.

Considering the formulated research questions we agreed that the semi structured interview format should be preferred.

We decided not to acquaint our research subjects with the original version of the questionnaire. We thought that familiarization with the instrument might restrain the respondents from disclosing their everyday life problems in full, especially those problems not captured by the RAQol.

The RAQol items were arranged into groups according to the dimensions of everyday life they reflect. Four groups emerged (note some overlapping): self-care and indoor activities (items 1, 3, 5, 7, 8, 10, 11, 18, 21, 26, 30), outdoor activities (items 2, 4, 6, 10, 14, 17, 20, 21, 25), emotions and conditions (items 9, 12, 13, 16, 19, 21, 22, 23, 24, 28) and relations (items 2, 4, 13, 15, 17, 20, 22, 25, 27, 29). We formed four open-ended interview questions to cover these dimensions. The questions were intended to introduce informal conversation during which different Qol aspects connected with the everyday life could be revealed.

1. How does your disease influence your coping with everyday indoor activities including self-care?

2. How does your disease influence your coping with outdoor activities?

3. What emotions can you describe in connection with your disease?

4. How has your disease influenced your relations with other people?

We prepared three to four additional secondary questions for each of the four interview questions for cases when some guidance of the interviewee is necessary.

The fifth interview question was added in order to give the interviewees a possibility to speak freely about their everyday life problems not assessed by the RAQol. The sixth question was intended to elucidate the hierarchical importance of everyday problems/restrictions for our patients.

5. What other impacts does your disease have on your life?

6. Which disease impacts on your life do you consider to be the most important?

We assumed the structure of the interview – from more detailed to general – to be appropriate for our patients; for most of them it would be the first time to be interviewed.

### The respondents

We were determined to include patients who met ARA 1987 diagnostic criteria for RA [14]. We decided to exclude patients with a concurrent disease or health condition, which, according to available medical documentation and the opinion of their physician, can be considered to have a significant impact on Qol.

By choosing interviewees from among the inpatients of the Rheumatology department of Tartu University Hospital, one of two specialized rheumatology centers in Estonia, we had access to sufficient medical data to follow the established inclusion and exclusion criteria. In Estonian rheumatology inpatient care is the dominating approach and it often also comprises some traditionally outpatient procedures. Recruiting inpatients gave us the opportunity to sample the whole spectrum of RA patients in circumstances where participation in the research would not interfere with their everyday routine. Also, conducting interviews under hospital conditions allowed us to create similar settings, free of major distractors, for each conversation.

Our sampling strategy derived from the wish to collect as multifaceted data as possible. We applied principles of theoretical sampling where information gathered during previous interviews determined the selection of subsequent respondents in order to get views from different positions. The final list of characteristics, which we considered to be important for guaranteeing versatility of the sample, included: age, gender, duration of RA, severity of RA (assessed by functional class and radiological stage), education, working status, marital status, members of family unit, and living conditions. The characteristics of the patients are presented in Table 1.

**Table 1:** Patients' characteristics. For radiographic stage and functional class estimation, data were collected from medical records; the Larsen-Dale [28] and Steinbrocker [29] classifications were used respectively.

ID	Gender	Age	Duration of RA, years	Radiographic stage	Functional class	Education	Working status	Marriage status	Living with	Living conditions
1.	Male	30	1	II	II	basic	not working because of the condition	separated	parents	flat in village
2.	Male	58	12	III	II	vocational	not working because of the condition	married	wife	flat in town
3.	Female	38	15	IV	III	vocational	not working because of the condition	married	husband and daughter (15 years old)	house in village
4.	Female	54	20	III	II	vocational	working	married	husband	house in village
5.	Female	66	10	V	II	secondary	retired	married	husband, grown-up daughter, grandson (11)	flat in town
6.	Female	49	4	IV	III	higher	not working because of the condition	single	female flat mate	flat in town
7.	Female	47	13	III	II	higher	half-time working	separated	son (13)	flat in town
8.	Male	54	10	IV	III	secondary	working	married	wife	farm
9.	Female	64	20	IV	II	vocational	not working because of the condition	widow	grown-up daughter	flat in town
10.	Female	69	50	V	III	higher	retired	separated	alone	flat in town

### The process of data collection

The interviewing took place from February to June 2002.

We consulted medical records to determine inclusion and exclusion criteria, patients' demographics and disease characteristics. No patients were contacted earlier than on the third day in hospital in order to allow them some time for adjustment. A day before the planned interview the goal and expected course of the interview were explained to the patient, the patient's were asked for their agreement to be interviewed. One of the patients we contacted refused to participate on account of being due to leave hospital the next afternoon. All recruited patients gave their informed consent.

Interviewing was conducted in a private room in the rheumatology department. All interviews were conducted by one researcher (MT) and were audio taped. Eight interviews were carried out in Estonian; two respondents (3 and 5) were interviewed in Russian, the respondents' native language. Interviews lasted from one and a half hours to three hours.

For most of our respondents it was their first chance to discuss their everyday life problems with somebody outside the family. But all of the interviewees were very willing to share their experiences after overcoming some diffidence at the beginning of the interview, and talked openly about their lives with the disease. For the interviewer, growing knowledge with every subsequent interview allowed her to move away from strict adherence to the interview questions, towards more informal conversation and, if necessary, the examination of some topics in depth.

Every interview was transcribed word-for-word and discussed before recruiting the next participant. Those interviews conducted in Russian were translated from the tape by the interviewer and transcribed in Estonian.

The tenth interview was exceptional. This patient was not hospitalized in the rheumatology department at that time, but we decided to invite her to our study due to the exceptionally long duration of RA recorded in her medical documentation – 50 years. The patient was contacted by phone and her agreement to participate was reached. We met at the patient's home and she was asked to tell her life story with stress on everyday problems. Thus we got an exiting narrative history of Estonian rheumatology from a patient's perspective and collected valuable information for our current research.

No new topics relevant to our research questions came forth after the seventh interview. Therefore we decided to stop at the tenth. This decision agrees with the theoretical sampling idea that one should stop recruitment of respondents when the researchers decide that the study has reached its saturation [15,16]. The intensive discussion we carried out simultaneously with the data collection gave us the opportunity to determine the stage when the inflow of new data no longer added any essential information to our study.

### Coding and analyzing the data

As the first step we read and discussed the interviews' transcriptions extensively. Tentative code families were chalked out as a notional framework for subsequent coding.

Open coding of the transcripts was performed independently by the two of us, MT and JS. The codes adhering to the previously identified code families were ascribed to expressions composed mostly of one or two sentences in order to distinguish their leading ideas; to some expressions several codes were attached Due to the different structure and extent of the tenth interview, selective coding was applied and only those parts relevant for our research questions were coded.

There were no disagreements between researchers at the level of code families; some inter-coder discrepancy appeared in appointing particular codes. Still, full consensus on open codes was reached in discussion. We analyzed the differences in coding and concluded that they could be ascribed to the coders' different – medical and sociological – backgrounds. We illustrate our conclusion with the example of coding a patient's expression: can't even go help my sister in the country with potato planting, a bit sad (2). It was coded as 'inability to offer physical help' by MT and 'alterations in traditional family relations' by JS; coders agreed on the 'relations with close ones' code family. During discussion the consensus code 'inability to perform in family roles' was established, which introduced a deeper exploration of the topic of role performance.

We agreed on the coding discrepancies being a benefit rather than a drawback of our research process. They allowed us to highlight and subsequently integrate different aspects of applied codes on the boundaries of marked tentative code families. Hence we decided not to perform formal inter-coder reliability analysis due to its diminished informative potential for this particular research.

Axial codes were created through grouping and condensing of open codes by MT. Axial coding was discussed and approved by all researchers. Side by side examination of affined axial codes of different interviews formed the basis for creating core categories – composition of a syllabus of motifs that emerged from interviews. Core categories were formed together by MT and JS, and were discussed and acknowledged by the whole research group.

In analysis we used the selective sampling of core categories to meet our two research objectives. First, to assess the importance of the RAQol topics for our patients, we compared every single item of the RAQol with the coded data of our interviews. Our assessment of importance was born in discussion and took account of the closeness of the meanings of the item and the relevant expressions of the interviewee, the frequency of their occurrence, and the significance for the respondents. Second, to describe the Qol topics that were significant for our patients but were not evaluated by the questionnaire, we compared the coded data without a counterpart among the items with the list of needs offered by the needs-based Qol model.

## Results

We will present our results as answers to the research questions. The descriptions of two of the three topic categories are included in the first, and the description of the third category in the second answer.

### The importance of the RAQol topics for our patients

Three groups of items can be highlighted: items whose importance was demonstrated by the data; items that could conditionally be considered to be important; items whose significance could not be shown on the basis of the interview data.

#### Important items

Most topics assessed by the instrument were essential for our interviewed patients. For 22 of 30 items, the interview data provided sufficient evidence to consider them applicable for the evaluation of the Estonian RA patients' Qol. Examples of patients' utterances supporting the significance of the content and also the appropriateness of the format of each of these items can be given from two or more interviews.

In some cases a remarkable diversity of utterances connected with one particular item was noted. We will illustrate this finding using item 17 as an example: I'm unable to join in activities with my family or friends. The following responses represent six different reasons to agree with the proposition included in the item. We have given word for word translations of the quotes into English, the IDs of patients are given in brackets.

##### Walking difficulties

• physically difficult to walk anywhere (1)

• a whole fuss with moving, don't want to torture myself (2)

##### Financial restrictions

• I used to ride the bus a lot before, now it's so expensive, I can't afford to visit anyone very often (9)

##### Difficulties connected with forced immobility

• Whenever you're sitting somewhere, are somewhere, it's hard to stay in one position all of the time, you have to make yourself move, go somewhere, or whatever (1)

• when your feet are ill and you sit for a very long time, then you can't even get up and move (4)

##### Changed quality of participation

• what's the use of going if you're no good anyway (2)

• of course I didn't go anywhere, only peeped from the car window (5)

• not going to the pub, don't know what to do there, can't handle dancing, and drinking doesn't work out either, no point in just sitting there the whole night (8)

##### Being ashamed of themselves

• when my joints were so tender and painful that I had to talk about my disease all the time then I definitely didn't look for company and I couldn't eat anyhow and I used a spoon for eating food that you ought to eat with a fork (9)

##### Unwillingness to create problems

• as I cause such a situation that my hosts have to help and watch me all of the time, I'd rather not go (6)

##### Worries about coping

• conditions of a home I go to, how cold it might be, if there's only cold water, is the toilet outside (6)

Difficulties related to bathing were mentioned in eight interviews and therefore item number 18: I have problems taking a bath/shower, was included in the group of the appropriate items. Still, the interviews also offered some hints that the value of the item as a measure of ability to carry out body care could be diminished by Estonian traditional sauna culture, especially popular in rural areas (more than one third of the Estonian population is rural.)

I don't care much for the bath, mainly I have let my kids take me to the country and have gone to the sauna, that's much more like it (2)

• sometimes I go to the sauna, we have a sauna in the cottage in the countryside, its no big deal to wash myself there, in the summer at least; a sauna might really be more convenient than this bath; there's just no hassle with getting up (7)

• I can manage washing myself in the sauna but I can't get up from the bath on my own (8)

#### Items that could conditionally be considered to be important

Motifs related to four of the remaining eight items emerged a number of times from the interviews. However, before adding these items to the list of appropriate ones, certain aspects should be taken into consideration.

Two respondents (2 and 5) burst into tears during the interview when looking back on their lives with the disease, which verified the significance of item number 19: I sometimes have a good cry because of my condition. The third interviewee spontaneously talked about crying, describing it as something bright and relieving. It can be presumed that in some cases crying may be interpreted as a way of coping, which is not unambiguously related to the level of Qol.

• if you cry, you should cry thoroughly and then you'll feel better, but if you keep something inside you it'll start eating at you, if you get a chance to have a good cry it'll make you feel good /.../ crying is self-purification, later you feel light; that's a feeling a healthy person doesn't get (9)

A single word or expression matching English 'frustration' cannot easily be found in Estonian. The foreign word 'frustratsioon' has been introduced into Estonian quite lately but remains unknown to the majority of lay people. Although there are a number of expressions in the interview data corresponding to item number 9: I often get frustrated, formulation of an Estonian version of the item suitable for embracing them all will be complicated.

• this is the feeling that you just are, the disease has already got a hold, the sequence of events just keeps going on and on (2)

• feelings of injustice that someone else is well but I'm not (5)

• it depresses me, and what the mind has built up is ruined by some moment and then the emotional breakdown comes again (6)

• often my thoughts run ahead of me and I'm feeling rested and would like to do something, but when I get down to it, then that's it, I end up hindering the work and not doing anything myself (8)

• I can't forgive my disease for ruining my life structure that had been carefully and arduously built over a long time (6)

In four interviews the insecurity connected with the disease progression and its unpredictability was one of the main topics. In Estonia's rapidly changing society, securing ones future cannot be an easy task and disabled people do not have much of a chance for success. Therefore the likening of capability to determine their future to ability to control their disease by patients is expected, and item number 28: I feel that I'm unable to control my condition, can be considered to be important. But again, difficulties in the formulation of the Estonian wording of the item will arise. Though quite common in everyday spoken Estonian the equivalent to the expression 'to control a condition' was never used by the interviewees when talking about their concerns.

• afraid of planning, you never know when it strikes back again (1)

• not yet [unable to cope] but it might come in the future; I don't know what will happen next year /.../ you're afraid that if you really get so poorly that you can't take care of yourself who will take care of you (4)

• I still try to be like a human being but don't know how long I can manage (5)

• there's really nothing for granted in this world of course, but I am preparing a back-up solution in case things get worse (8)

No patients reported that they were disturbed by continuously thinking about the disease. Three interviewees with a disease duration of over 10 years talked about not thinking of their condition as a positive phenomenon connected with adaptation to the disease. We concluded that item number 23: My condition is always on my mind, can be treated as significant, although our interviews revealed no evidence that the problem is recognized when present.

• I don't think that I'm an ill person at the moment, just when these hands are painful or I just can't cope with everything; I don't think that I'm ill, that I'm so miserable, I don't think about it (4)

• I have to change and re-adjust my basic values, but I don't think about it all of the time, maybe I have adjusted them subconsciously /.../ I then, subconsciously, not thinking about it, eat something softer or don't go biting on a big apple if my jaw joint is painful (7)

• I don't' even think about my disease at this point, it's like a husband now, day and night, its there in everything I do and I know that as long as I live I will have it and I cant get rid of it and I don't make it a problem anymore (9)

#### Items, whose significance could not be shown

To this group we included four items.

In one interview, the impact of pain on attention was described. We did not find this evidence sufficient for designating item number 12: I find it hard to concentrate, as significant. In our opinion, these expressions describe switching of attention to another stimulus and do not refer to concentration difficulties. One reason why the interview data did not support the importance of this item can be the lack of currently active intellectual workers in our sample.

• You can't think about anything but pain; crossing the road you might get hit because you're only thinking about the pain and forget that you have to keep an eye on the road (6)

No data corresponding to item number 1: I have to go to bed earlier than I would like, emerged from the interviews. Because tiredness is a characteristic feature of RA, one explanation for the inability of our data to show this item's importance is associated with the paucity of absorbing nighttime activities, especially in Estonian rural areas.

Item number 24: I often get angry with myself, had no matches in our interviews. It is difficult to find any obvious explanations for this particularity but we can suggest that in some cases anger was rechanneled against the medical system – a phenomenon that we will discuss later.

Our data failed to demonstrate the importance of item number 28: I avoid physical contact. We believe that intimacy connected with this topic could be the reason our respondents avoided openly discussing it. If so, due to the greater impersonality of a questionnaire format, the item can retain its significance as a part of the instrument.

### What else our patients found to be significant in connection with their everyday life quality

As a result of the analysis, three groups of topics connected with satisfaction of the needs included in the list of those crucial for Qol and important for our patients, but not assessed by the questionnaire, were described.

Next we will name these needs and present the evidence of the limitations in their fulfillment. Our deeper inquiry into the reasons for these peculiarities will be presented in the discussion section.

#### Identity, status, appreciation, respect, usefulness to others, and self esteem

Adaptation to a disease is a difficult process comparable to passing through phases of grief. An inevitable and hurtful part of it is abandoning of old roles and the recognition of new ones.

Changing role functioning was a common motif in the interviews. Regret and anxiety due to inability to perform in the roles of a healthy person were expressed by the majority of respondents.

##### Gender role

• What man isn't disturbed by the inability to take care of himself, then you're like a kid not a man (8)

• My appearance isn't as attractive [as a woman] anymore as it could have been without the disease (4)

##### Role in family

• I can't even go help my sister in the country with potato planting, a bit sad (2)

• just sad because of him [the son], he asks me why I can't come outside to play soccer with him, well, I really can't (7)

##### Age role

• totally like a small kid, someone else has to help you all the time, whatever it is, meals or something else (1)

• I walked with a stick, it was a catastrophe, such an old granny (3)

##### Work related role

• I want to do something, just to make something and do a job, my hands want to work; people drop by and try to rope you in – I can't, there's no doer, all the time "I can't", other days I can't at all (2)

• I am afraid of going to work soon; this hand is so ugly; what a hairdresser with such a horrible hand! (4)

The new role of a diseased person was generally interpreted as something deprecatory; being ill was considered as opposite to being normal.

• I can't move; my movements aren't like they should be, not like healthy and normal people have (1)

• before I used to wear high heel shoes like normal people (3)

• you're like some prehistoric creature, everybody goes by modern means but you like going back to the stone age (6)

Feeling ashamed of the disparity led to preoccupation with concealing the condition. Surrounding people were often seen as appraisers; their opinions were valued more highly than success in coping was. As a result, even the simplest aids that could be noticed by others were rejected. In some cases the fear of being labeled as different elicited the preference of social isolation.

##### Concealing the condition

• I don't want to see things that point to my condition, don't want these to be seen, and of course, don't want to have to always hide everything (6)

• I control myself so that others won't notice the way I am – I have so many acquaintances that for a long time didn't know I was ill (9)

• I'm even afraid to tell anyone that this hand is so ill, I'm so quiet (4)

##### Rejection of aids

• [a special cup with two ears] I have it at home, but I don't use it; at this point I still try to be humanlike (5)

• you don't go shopping with a crutch, no way (8)

• I feel very uneasy eating in company; I still want to eat like people do, with regular tableware (9)

##### Preference of social isolation

• if I still find it impossible to eat or drink in company, then I don't; I won't go into company [to eat] like this (6)

#### Safety, freedom from fear, and stability

A well functioning medical system should strengthen the feelings of security and stability of its clients. In the words of our respondents the medical system constituted an enemy. The system was something to blame, to vent anger on; at the same time it was described as an inevitability that had to be obeyed and against which protection was required.

##### Blaming

• the system is wrong, the sick funds and all, why can't I get procedures done that are necessary for me (3)

• you sign up there [for a rheumatologist], you wait and wait, a lot changes in that time; you wait a month and a half; you get there; by that time the drugs have run out, later you're at fault for not having taken them (2)

• I went to a private clinic, almost like crying at the door, there are no vacancies, there's no one I could talk to, and I didn't. I went away to the country/.../now I'm here and now I'm told that this Achilles' has been broken for at least a month (10)

##### Obeying and need for protection

• [left alone with the disease] just can't be such a thing, not alone, but life is like this, don't know what to wish (2)

• helplessness, no person to protect me [against the system] (3)

Physicians, some of them named as saviors and supporters, were still more often seen as being an impersonal part of the system. Professional incompetence and superficiality were connected with *non grata *turns in the course of the disease. One of the respondents regarded physicians as co-victims of the system, incapable of defending their patients.

##### Part of blamed system

• if I had been sent to the rheumatologist right away then maybe things wouldn't have gotten this bad; thus, when my neck was stiff at first, it was thought I had caught a cold and it would pass (1)

• the troubles started when my leg trauma was labelled as radiculitis (2)

• initially no action was taken and consequently the general attitude [of the doctors] became such that nobody had the guts to do anything with me, and because it was such a awkward situation because they didn't have the right doctor who would have done something, I was left a bit high and dry (10)

##### Co-victims

• the kidney doctor said: not to come to me, I am out of money, go to the GP /.../you go to the GP, she can't help you, prescribes what she can, she can't prescribe rheuma-drugs (2)

#### Procuring food, drink and other necessities of life

The ability to do necessary shopping is directly assessed by item number 6: I find it difficult to walk to the shops. Difficulties with walking to the shop were described by our respondents and the item was considered to be important. But other problems connected with shopping emerged even more often in the interviews – managing in the shop, checking out, and carrying the purchases.

##### Managing in a shop

• I don't want to stand in a line, my feet start to ache (7)

• [shopping trolley] is very big, you have to push it hard and it's narrow there [in the shop] (9)

##### Checking out

• checking out at the counter you're in a hurry and then comes this psychological moment that you get nervous and can't handle it (7)

• I open my wallet, they take what they need (5)

##### Carrying the purchases

• when the bag is too heavy then my feet can't stand it; you have calculate how much you buy (6)

• sometimes you would like to buy more but you can't carry it; I just put stuff that I really need in the cart (9)

For our interviewees, a topic closely related to shopping was the capability to use public transport. The obstacles encountered when entering, leaving and riding a bus were described.

##### Getting on

• I can't get onto the bus, even worse with the tram; the steps are very steep, I can't manage however I try (3)

##### Getting off

• I can't get off, I ride to the next stop; the bus doesn't pull over to the sidewalk but stops further away (6)

##### Riding

• very bad to ride, I just stagger, lose my balance, sometimes you can fall quite badly; the worst is when you have to stand (9)

• sitting down and getting up [on bus] is difficult for me, it's better I hold on to something for some time (5)

The lack of money was mentioned by all of our respondents. Its interference with the satisfaction of the majority of needs was described.

• I wouldn't say my spending has increased a lot, but my income is, yes, notably lower; before I even paid more income tax than I get paid now; the habits I had before have become impossible for me, financially (1)

• a handicapped person could also make his/her life comfortable and tolerate everything if it was possible financially (6)

• the pension is so tiny, I can't do anything with this; I can't manage a family with this (7)

## Discussion

Our results showed the high significance of the majority of the RAQol items for the interviewees. This allows us to state that the RAQol can successfully be adapted into Estonian for usage in international research projects. Our results also highlight the difficulties in translating some specific items (number 9 and 28), which should be reckoned with during the adaptation process.

Three Qol aspects that were important for Estonian RA patients but were not evaluated by the RAQol – the issues concerning changes in role performance, safety and stability of communication with the medical system, as well as some issues of procuring the necessities of life-were revealed by the analysis of interviews.

Next we would like to discuss the reasons for these peculiarities and suggest some additional resources for in-depth reading.

Only 13 years have elapsed since the end of Soviet rule in Estonia. Our respondents spent a considerable part of their lifetimes in the Soviet ideological environment and this has undoubtedly influenced their values and beliefs. An obligatory component of the *homo sovieticus *mentality was the placing of collective interests above personal ones; individuals were appreciated by their contribution to the common good. Successful performance in social roles approved by the regime was honored; inability to meet validated ideals was considered shameful. Although we can talk of a dramatic change of approbated values and ideals after the end of the Soviet era – an independent, successful, competitive young individual is idealized now –, but the tendency to disapprove of the inability to fit expectations has remained.

We believe that this potpourri of old and new values and attitudes could explain the high significance of themes connected with roles and role functioning in our respondents' conversations. A person disabled because of disease could not perform successfully in acknowledged Soviet-time roles (the existence of people with special needs was simply hushed up by the Soviet media); the same is true for present-day Estonia. Being categorized as "socially uncompetitive" would alter the identity and self-esteem of the disabled individuals and concealment of the condition could be seen as defense reaction. In the light of this, topics connected with role functioning and social acceptability should be included among those observed during a Qol investigation. See also [17-21].

The transition from one economic and ideological system to another in Estonia has caused social instability, which is reflected in an increase in people's subjective sense of insecurity and fear. The most insecure time for the Estonian population was in the early 90ties; since that time, due to stabilization of the economy and economic development, a sense of security is returning. Still, there is remarkable disaffection with different spheres of public administration; future-oriented reorganizations that do not provide immediate benefits are treated with caution. The health care system, which has undergone fundamental reforms in the post-soviet period, is the favored object of criticism for the Estonian media. In the Soviet era health care was funded from the state budget and all citizens had free access to health services. Today's health care delivery system in Estonia is financed through health insurance; the private sector is growing. The introduction of family practitioners, with novel financing principles and responsibilities, in the mid 1990ties has changed previously existing relations between patients and medical specialists; access to consultants has lost its immediacy. Bureaucracy, long waiting lists, visit charges (something unthinkable for Soviet medicine) and open discussion of the financial difficulties of the health service in the Estonian media have all made their contributions to lowering confidence in the health care system in the eyes of our patients. Communication with health care deliverers constitutes a significant part of everyday life for RA patients. Therefore the impact of this communication on the everyday life of patients cannot not be ignored. In the case of a transitional society like Estonia's, the effect of health care delivery on patients' needs for stability and safety should be considered. See also [22-26].

In health care planning, the strategic military interests of the Soviet Union were given priority; this resulted in the development of an excessive large hospital network. Habilitation and rehabilitation were upstaged and drastically under-funded. In the Soviet era the people with special needs were partly institutionalized as disabled; their problems and even their existence (with the exception of war veterans) were simply hushed up by the authorities and media. There were no adaptations for people with physical special needs in public places and in the physical environment of towns in Soviet Estonia; elementary facilities were not accessible. People in wheelchairs began to be seen on our street with arrival of western tourists in early 90ties; they were joined shortly afterwards by our own special needs persons. During the following years adaptations considering the requirements of people with special needs have little by little been introduced into the physical environment. However, due to limited resources the improvements are coming slowly and conditions have not reached the level where the comfort of special needs persons can be guaranteed. Coping with errands and chores should be taken into account as a distinct Qol topic in Estonia today. See also [27].

According to the Statistical Office of Estonia  the average monthly pension for the disabled in Estonia in 2003 was 1110 Estonian kroons (approximately 71 EUR). This constituted one sixth of average monthly gross wages and salaries, and was 301 kroons (19 EUR) lower than estimated minimum means of subsistence for 30 days in the same period. From these figures we can see that financial troubles can overshadow every aspect of the everyday life of our patients.

In our opinion adding the assessment of these three aspects – changes in role performance, safety and stability of communication with the medical system, and some aspects of procuring the necessities of to life – should be considered by Estonian researchers, especially when carrying out disease-specific Qol studies at the national level.

## Conclusions

In our research we used qualitative interviews for assessing the appropriateness of a RA-specific Qol instrument, the RAQol, for adaptation for use in Estonia. We described the importance of the items to our patients and identified Qol topics that were significant for our respondents but that were not assessed by the questionnaire. We also discussed the nature of the discrepancies in significance of Qol topics for Estonian patients.

Our results show that the utilization of a qualitative study as an introductory part of Qol assessment instrument adaptation provides possibilities for more thoroughly considered Qol research. By evaluating the significance of items in the particular context, it allows the mechanical acceptance of instruments just because they have performed well in other societies and cultures to be avoided. Moreover, it offers an opportunity to identify topics that are not included in the instrument but are important for local interpretation of Qol, which are otherwise often overlooked. For researchers, qualitative studies offer a deeper understanding of the instrument in question and of the research topic – Qol of the patients.

The data collected during this qualitative research process also has the potential to be used for a wider analysis of the Qol of Estonian RA patients. Still, our current interests were centered on the appropriateness of the adaptation of a particular Qol instrument, and therefore the use of the gathered data was quite limited. But we believe that the knowledge gained will be beneficial for our forthcoming research projects.

## Authors' contributions

MT: the idea and conceptual construction of the research, recruitment of the participants and conduction of the interviews, coordination of the coding and analysis processes

JS: methodological construction and supervision of the research, active participation in the coding and analysis processes

KM and EH: participation in discussions and decision-making throughout the whole course of the research

All authors have read and approved the final manuscript.
